# Transcriptome sequencing identifies ANLN as a promising prognostic biomarker in bladder urothelial carcinoma

**DOI:** 10.1038/s41598-017-02990-9

**Published:** 2017-06-09

**Authors:** Shuxiong Zeng, Xiaowen Yu, Chong Ma, Ruixiang Song, Zhensheng Zhang, Xiaoyuan Zi, Xin Chen, Yang Wang, Yongwei Yu, Junjie Zhao, Rongchao Wei, Yinghao Sun, Chuanliang Xu

**Affiliations:** 1 Department of Urology, Changhai Hospital, Second Military Medical University, Shanghai, P.R. China; 2 Department of Geriatrics, Changhai Hospital, Second Military Medical University, Shanghai, P.R. China; 3 Department of Pathology, Changhai Hospital, Second Military Medical University, Shanghai, P.R. China

## Abstract

The prognosis of bladder urothelial carcinoma (BLCA) varies greatly even for patients with similar pathological characteristics. We conducted transcriptome sequencing on ten pairs of BLCA samples and adjacent normal tissues to identify differentially expressed genes. Anillin (ANLN) was identified as a transcript that was significantly up-regulated in BLCA samples compared with normal tissues. Prognostic power of candidate gene was studied using qRT-PCR and immunohistochemistry on 40 and 209 patients, respectively. Patients with elevated ANLN expression level was correlated with poorer cancer-specific (median, 22.4 vs. 37.3 months, p = 0.001), progression-free (median, 19.7 vs. 27.9 months, p = 0.001) and recurrence-free survival (median, 17.1 vs. 25.2 months, p = 0.011) compared with low ANLN expression. Public datasets TCGA and NCBI-GEO were analyzed for external validation. Knockdown of ANLN in J82 and 5637 cells using small interfering RNA significantly inhibited cell proliferation, migration, and invasion ability. Moreover, knockdown of ANLN resulted in G2/M phase arrest and decreased expression of cyclin B1 and D1. Microarray analysis suggested that ANLN played a major role in cell migration and was closely associated with several cancer-related signaling pathways. In conclusion, ANLN was identified as a promising prognostic biomarker which could be used to stratify different risks of BLCA.

## Introduction

Bladder urothelial carcinoma (BLCA) is the sixth most common cancers in North America, and the sixth leading cause of cancer-related deaths in Europe^1, 2^. The stage and grade of BLCA are strongly associated with prognosis and play an important role in determining treatment modality^3^. However, the risks of disease recurrence and progression remain significant variability in patients with similar pathological characteristics. A third of non-muscle invasive bladder cancer (NMIBC) patients may later progress to muscle-invasive (MIBC) or metastasis, and around 70% of patients who undergo radical cystectomy and lymphadenectomy progress to metastatic disease^4–6^. As a result, identification of prognostic molecular markers is critical to help urologists in the stratification of high- and low-risk BLCA patients to make individualized treatment decisions^7, 8^. Next-generation sequencing technologies have become a powerful tool for comprehensive characterization of point mutations, copy number alterations and changes in gene expression^9–11^. Transcriptome sequencing (RNA-seq) of tumors is an efficient approach to identify molecules involved in carcinogenesis and reveal biomarkers with prognostic value^3, 12^.

Anillin (ANLN) is an actin-binding protein, which is firstly identified in *Drosophila* and primarily involved in cytokinesis^13, 14^. ANLN is capable of recruiting several key cell division-related components, e.g. F-actin, myosin II and septins, to the cleavage furrow during cytokinesis and is recognized as the central organizer at the hub of the cytokinetic machinery^15^. Dysregulated ANLN expression has been found in a wide variety of human cancers, i.e. breast, colorectal, endometrial, liver, lung, renal, kidney, ovarian, and pancreatic cancer^16^. ANLN is up-regulated from 2 to 6 fold in tumor tissue, except for brain tumors, and its expression increases from normal to benign to malignant to metastatic disease. ANLN thus possesses great potential as a biomarker of cancer progression^14, 16, 17^. However, the underlying role of ANLN in BLCA has not yet been elucidated.

Here, we performed RNA-Seq analysis on ten pairs of BLCA samples and their matched adjacent normal tissue. We observed a number of differentially expressed genes common to the ten pair samples. Among these genes, we found ANLN expression was correlated with pathological stage and was a promising prognostic biomarker. Functional studies further demonstrated that ANLN participated in the regulation of bladder cancer cell proliferation, migration and invasion, along with the cell cycle. Moreover, the mechanisms responsible for these effects were further explored using microarray analysis. Taken together, our data suggested that ANLN was a novel and promising prognostic biomarker for BLCA that may aid in the risk stratification.

## Results

### Differential gene expression profiling revealed by RNA-seq

**Figure 1:**
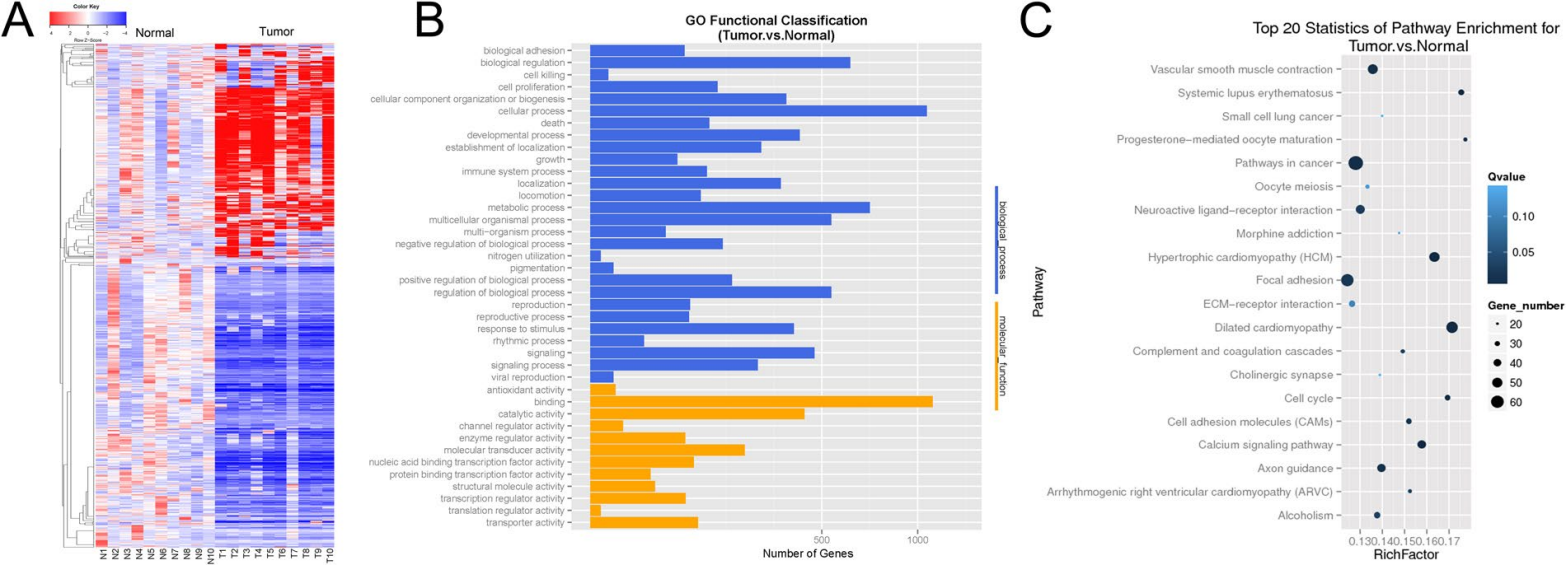
Transcriptome sequencing expression profiles in bladder cancer and corresponding noncancerous tissues. (**A**) Hierarchical clustering heat map of expression profiles for genes with log_2_-fold change >1 and probability of >0.6 (1516 genes). (**B**) Enriched Gene Ontology (GO) biological process and molecular function. (**C**) Top 20 significantly changed pathways.

RNA-Seq was performed on 10 pairs of matched tumor and adjacent normal tissues from patients with BLCA. The cutoff of log_2_-fold change >1 and probability >0.6, a method proposed by Tarazona *et al*.^18^ which was more effective in controlling the rate of false discoveries, was used to filter genes that were differentially expressed between BLCA samples and the matched normal controls. With both criteria, a total of 1516 (676 up-regulated and 840 down-regulated) out of the 22,162 expressed genes were selected. Differential expression patterns of these genes were shown in hierarchical clustering heat map (Fig. 1a). The same gene set was used for Gene Otology (GO) terms and pathway analysis. GO analysis was performed using GO:TermFinder (http://go.princeton.edu/cgi-bin/GOTermFinder), and significant biological process and molecular functions were shown in Fig. 1b. Pathway analysis was performed on the same gene set using DAVID (https://david.ncifcrf.gov/) pathway analysis tool. A selection of the top 20 statistically significant pathways were shown in Fig. 1c, several aberrant pathways were closely associated with carcinogenesis, e.g. pathways in cancer, cell cycle and focal adhesion.

### Identification of ANLN as a potential prognostic gene in BLCA

**Figure 2:**
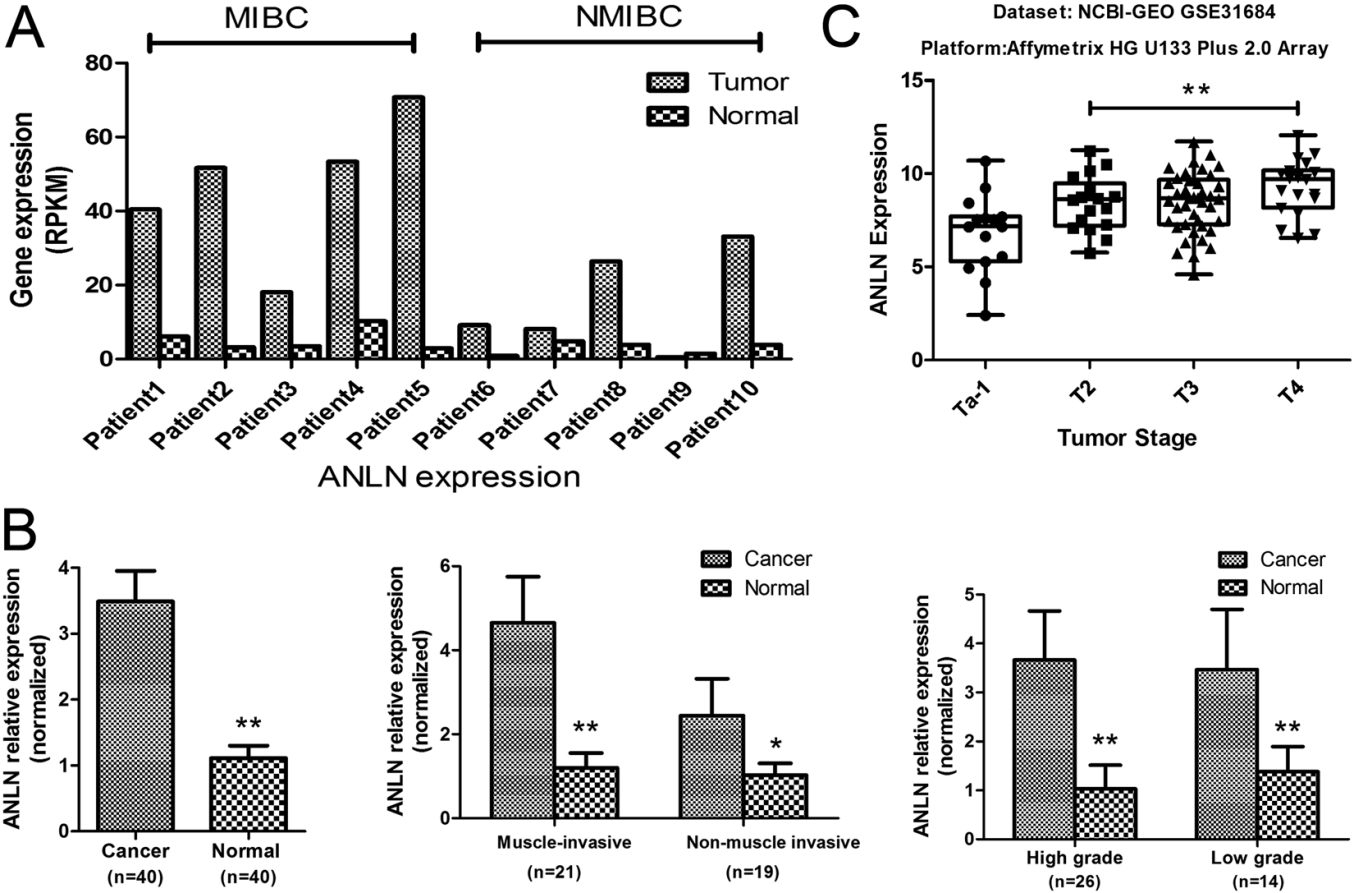
ANLN was overexpressed in tumor tissue and its level increased with bladder cancer stage. (**A**) Expression levels (RPKM) of ANLN in each sample for transcriptome sequencing. (**B**) ANLN expression was examined by qRT-PCR in 40 paired bladder cancer and adjacent noncancerous tissues. (**C**) ANLN expression in different stages of bladder cancer using the NCBI-GEO GSE31684 dataset. MIBC = muscle-invasive bladder cancer; NMIBC = non-muscle invasive bladder cancer. *p < 0.05; **p < 0.01.

ANLN was identified as a candidate gene of interest for three reasons. Firstly, ANLN expression was up-regulated, with a log_2_-fold change of 2.926 and probability of 0.835, in tumor samples compared with corresponding normal tissues. The expression level, quantified by reads per gene per kilobase exon per million mapped reads (RPKM), was up-regulated in 9 out of the 10 tumor samples as shown in Fig. 2a. Secondly, ANLN was a potential biomarker for tumor staging due to its expression level increased with BLCA stage (Fig. 2b). Thirdly, after data-mining and literature search, ANLN was not previously reported to be associated with BLCA. ANLN expression pattern was further validated by qRT-PCR in an additional 40 paired BLCA samples. ANLN expression was confirmed to be up-regulated in the majority of tumor samples (36/40), especially in MIBC, when compared with their corresponding normal tissues (Fig. 2c). Given the increased expression level of ANLN was observed in MIBC, we analyzed NCBI-GEO datasets to determine whether the similar trend was also evident in publicly available resources. As indicated in the GSE31684 dataset, published by Memorial Sloan-Kettering Cancer Center with 93 patients with high-risk BLCA, the ANLN expression level, on average, significantly increased as tumor stage progress (ANOVA test, P = 0.006, Fig. 2c).

### ANLN was frequently overexpressed in BLCA and was correlated with prognosis

**Figure 3:**
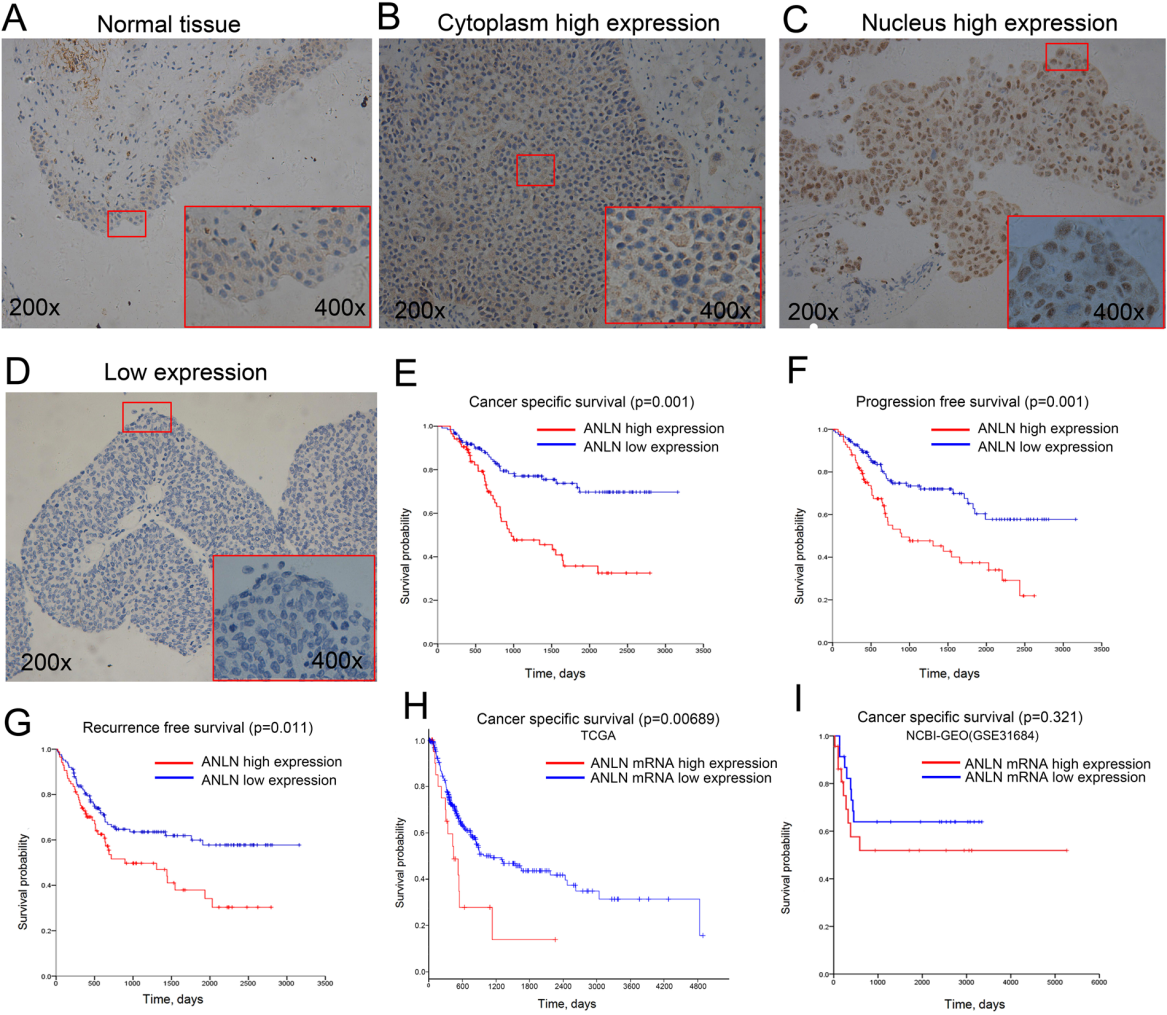
ANLN immunochemical staining revealed elevated ANLN expression as clinically relevant. (**A**–**D**) Representative images of ANLN expression in normal and bladder cancer tissues. (**C**–**G**) Higher ANLN expression was found to be associated with poorer cancer-specific survival, progression-free survival and recurrence-free survival from our single institution cohort. (**H**,**I**) Kaplan–Meier survival curve comparing cancer-specific survival time between different expression level of ANLN mRNA in TCGA and NCBI-GEO (GSE31684) datasets.

To investigate whether ANLN protein was overexpressed in BLCA and could serve as biomarker for prognosis, we evaluated its expression by immunohistochemical analysis in 209 patients who underwent cystectomy or transurethral resection of BLCA. Demographic and characteristics of the 209 patients were shown in Supplementary Table S2. ANLN expression level was always lower in normal urothelium that could be studied adjacent to tumor tissue (staining score,1.21 ± 0.59) than in tumor tissues (staining score,3.57 ± 1.1, p < 0.001, Fig. 3A,B). Of the 209 BLCA samples, ANLN expression was classified as low in 124 samples and high in 85 samples (Fig. 3B–D). Meanwhile, ANLN expression was significantly higher in MIBC and high grade tumors (p < 0.001, Supplementary Table S2).

With a median follow-up of 27.9 months, patients with high ANLN expression in tumor showed significantly poorer CSS (median, 22.4 vs. 37.3 months, p = 0.001), PFS (median, 19.7 vs. 27.9 months, p = 0.001) and RFS (median, 17.1 vs. 25.2 months, p = 0.011) compared with patients with low expression (Fig. 3E–G). In univariate analysis, CSS was significantly influenced by tumor stage, grade and ANLN expression level (Supplementary Table S4). In multivariate analysis, tumor stage (HR = 2.04, 95% CI: 1.33–4.8, p = 0.005) and ANLN expression level (HR = 2.04, 95% CI: 1.21–3.43, p = 0.007) were independent risk factors of CSS (Supplementary Table S4). Survival analysis was further performed individually on MIBC (n = 130) and NMIBC (n = 79) cohorts. In MIBC cohort, patients with high ANLN expression had a significantly reduced CSS (p = 0.012) and PFS (p = 0.018), while the RFS (p = 0.144) did not reach the level of statistical significance (Supplementary Fig. S1). Regarding NMIBC cohort, patients with high ANLN expression showed a tendential correlation with poorer CSS, PFS and RFS, but none of these parameters reached the level of statistical significance (Supplementary Fig. S1).

To validate these observations in our single institution, we analyzed the association between ANLN mRNA expression and clinical outcome in TCGA via CBIOPORTAL (http://www.cbioportal.org/). ANLN was defined as significant up-regulated with the threshold of *Z*-score above 2. There were 30 (7%) out of 408 patients with significant up-regulated ANLN mRNA and they had significantly poorer CSS (median, 14.75 months) compared with those who did not significant altered (median, 36.86 months, p = 0.007, Fig. 3H). Regarding the GSE31684 dataset from NCBI-GEO, patients with ANLN mRNA expression above 75% (n = 23) showed tendentially reduced CSS compared with ANLN below 25% (n = 23, median 10.8 vs. 65.3 months, p = 0.321, Fig. 3I).

### Knockdown of ANLN inhibited BLCA cells proliferation, migration and invasion

**Figure 4:**
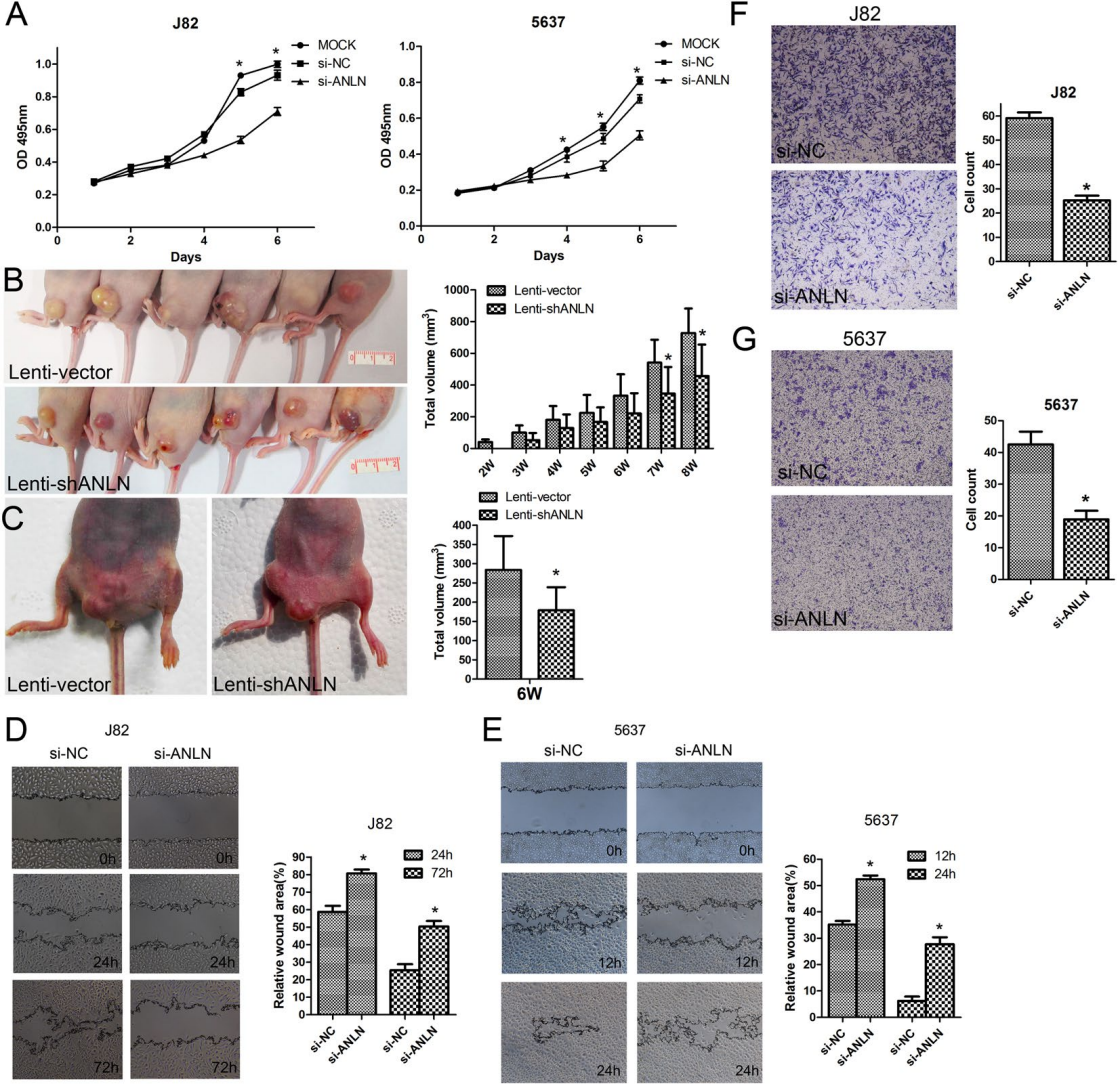
Knockdown of ANLN expression markedly inhibited bladder cancer cells proliferation, migration and invasion. (**A**) siRNA knockdown of ANLN in J82 and 5637 cells showed significant inhibition of cell proliferation in CCK8 assay. (**B**,**C**) The effect of ANLN knockdown on tumor formation in subcutaneous and orthotopic nude mouse models. Lenti-shANLN and vector-transfected J82 cells were used to establish the two mouse models. (**D**,**E**) Knockdown of ANLN significantly reduced J82 and 5637 cells migration in scratch migration assay. (**F**,**G**) Matrigel invasion assay demonstrated that knockdown of ANLN significantly inhibited invasion ability of J82 and 5637 cells. *p < 0.05; **p < 0.01.

To explore the functions of ANLN in BLCA, we used small interference RNA (siRNA) induced knockdown of ANLN expression in 5637 and J82 cells, which had relative higher ANLN expression among several BLCA cell lines (Supplementary Fig. S2). As shown in Fig. 4A, knockdown of ANLN could significantly inhibit the proliferation of J82 and 5637 cells. This finding was further confirmed by using *in vivo* subcutaneous and orthotopic nude mouse models, in which the tumor growth rate was significantly decreased after ANLN knockdown in J82 cells by using lentivirus-mediated gene transfer method (Figs 4B,C and S2). Cell migration and invasion were critical steps during cancer progression. The result of the scratch/wound-healing assay showed that ANLN knockdown reduced migration ability of J82 and 5637 cells (Fig. 4D,E). Furthermore, cell invasion ability of J82 and 5637 cells, as monitored by matrigel invasion assay, was also significantly alleviated after knockdown of ANLN in J82 and 5637 cells (Fig. 4F,G). Taken together, these results indicated that ANLN played an essential role in the carcinogenesis of BLCA.

### Knockdown of ANLN induced cell cycle arrest and abnormalities during binucleation

**Figure 5:**
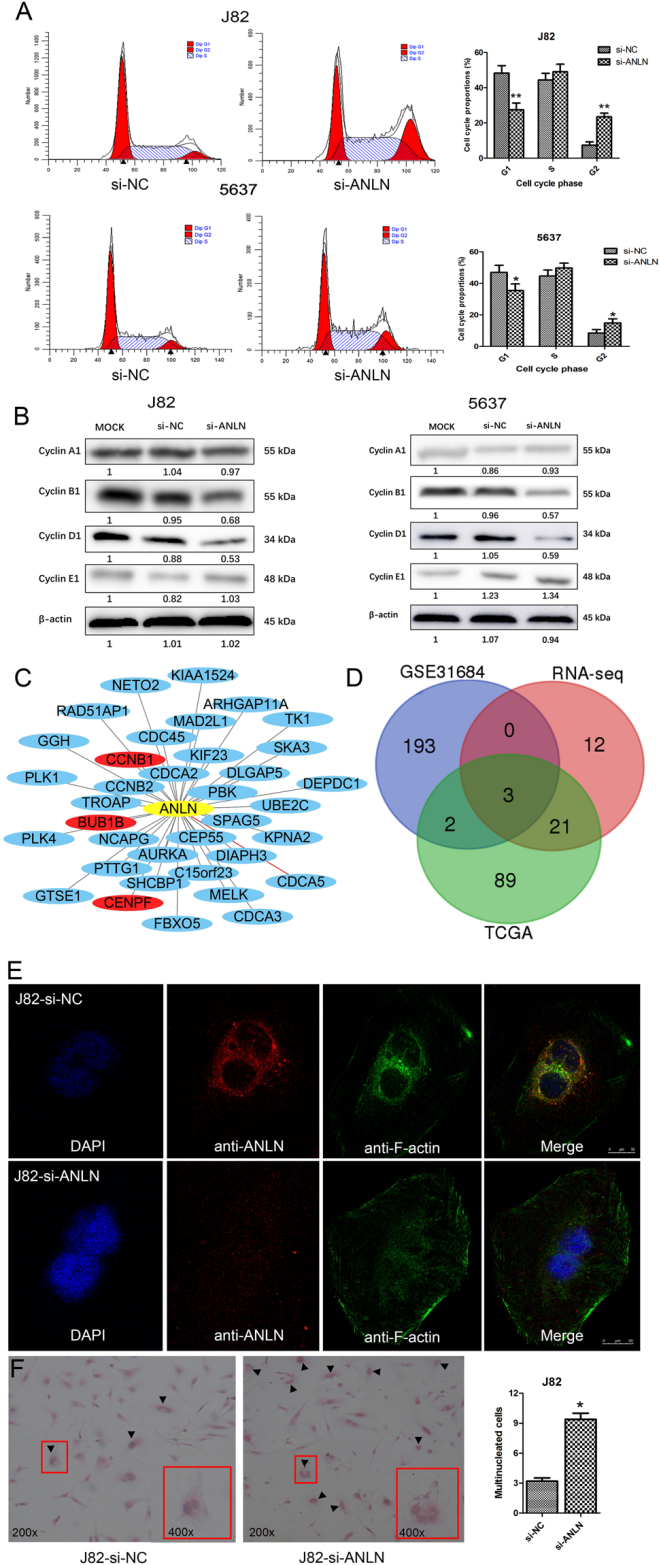
Cell cycle progression and binucleation process were impaired after ANLN knockdown. (**A**) Knockdown of ANLN induced G2/M phase arrest in J82 and 5637 cells. (**B**) Western-blot analysis of cell cycle checkpoint proteins in J82 and 5637 cells with different treatments (MOCK, si-NC, si-ANLN). (**C**) Network of ANLN co-expressed genes from present RNA-seq data, the distance between ANLN and gene nodes was negatively correlated with Pearson score (red gene nodes represented genes co-expressed with ANLN in RNA-seq, TCGA and NCBI-GEO datasets). (**D**) The Venn diagram of differentially expressed genes in three datasets. (**E**) Confocal images of ANLN and F-actin distribution after transfection of J82 cells with si-ANLN or control RNA. (**F**) Hematoxylin & eosin staining showed that the percentage of multinucleated cells significantly increased after knockdown of ANLN in J82 cells. *p < 0.05; **p < 0.01.

ANLN was reported to play an essential role in cytokinesis^15, 19^. To elucidate the regulatory mechanism of ANLN involved in tumor growth, we further performed flow cytometry to compare apoptosis and cell cycle phases between J82 and 5637 cells with ANLN knockdown and control cells. In terms of apoptosis, we found no significant difference between ANLN knockdown group and control group in J82 and 5637 cells (Supplementary Fig. S3). With regard to cell cycle analysis, treatment with si-ANLN significantly increased the percentage of G2/M phase compared with control cells, which suggested that cells with ANLN knockdown were arrested at the G2/M phase (Fig. 5A). Furthermore, we investigated the expression of cell cycle associated proteins 72 hours after ANLN knockdown. As shown in Fig. 5B, knockdown of ANLN resulted in reduced expression of cyclin B1 and cyclin D1 in both J82 and 5637 cells, while no obvious change of cyclin A1 and cyclin E1 was observed.

To find the key partner genes that may co-expressed with ANLN, we extracted genes that correlated with ANLN and had a Pearson score >0.6 or <−0.6 from RNA-seq data in the present study (Fig. 5C), and from TCGA and NCBI-GEO (GSE31684) datasets as well (Supplementary Table S5). As shown in the Venn diagram (Fig. 5D), three genes (CCNB1, CENPF and BUBIB) were found to be co-expressed with ANLN in all three datasets. Interestingly, all these genes were reported to be associated with mitosis and played an important role in the G2/M cell cycle phase^20–22^. The above results suggested that ANLN played a crucial role in the regulation of cell cycle in bladder cancer cells.

Immunofluorescence analysis showed that less ANLN protein was observed to form the contract ring for nuclear division in J82 cells with ANLN knockdown (Fig. 5E). This was consistent with previous results that ANLN played an important role to form a contracting ring during mitosis^13, 15^. J82 cells with ANLN knockdown and control cells were further stained by hematoxylin & eosin. Compared with control cells, multinucleated cancer cells were observed at higher frequency after ANLN knockdown (Fig. 5F).

### Gene expression alteration after knockdown of ANLN in J82 cells

**Figure 6:**
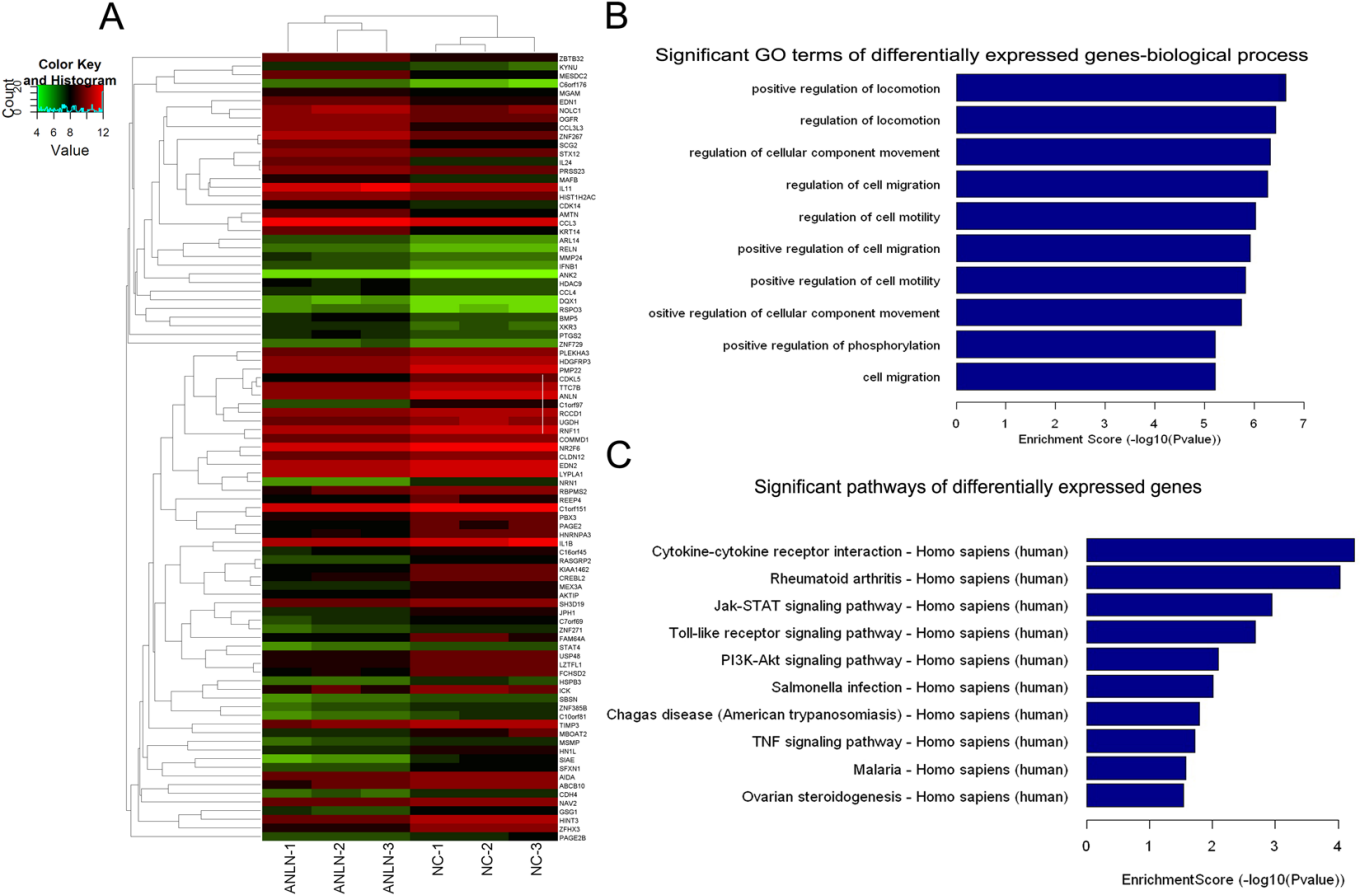
Changes of the gene expression profiling after knockdown of ANLN in J82 cells. (**A**) Hierarchical clustering heat map of the significantly dysregulated genes in 3 paired samples. (**B**) The top ten most obvious changes in GO biological process classification confirmed that ANLN was closely correlated with cell migration and locomotion. (**C**) Top ten enriched pathways for significant dysregulated genes.

To obtain a global perspective on how gene expression profile changed after knockdown of ANLN in BLCA cells, we performed microarray analysis to compare the ANLN knockdown group and control group in J82 cells. At cutoff of log_2_-fold change >1 and p < 0.001, 34 significantly up-regulated and 57 down-regulated genes in ANLN group compared with corresponding control group were selected for GO analysis and pathway analysis (Fig. 6A). As shown in Fig. 6B, most of the top ten terms in GO biological process classification were closely related to regulation of locomotion and cell migration, which was in line with the ANLN functional studies. The top ten most significantly dysregulated pathways were shown in Fig. 6C. Among several tumorigenesis related pathways, PI3K/Akt pathway was most representative, because ANLN nuclear localization and stability had been reported to be regulated by PI3K/Akt signaling pathway^23^. The specific altered genes related to cell migration and tumorigenesis related signaling pathways were listed in Supplementary Table S6.

## Discussion

In the present study, RNA-Seq analysis of ten pairs of BLCA samples identified 1516 genes that were differentially expressed between tumor and normal tissue. Pathway and GO analysis indicated that these genes were significantly associated with carcinogenesis processes such as pathways in cancer, cell cycle, focal adhesion, cell proliferation, providing important clues for understanding the pathogenesis of BLCA. Among these genes, we identified ANLN, overexpressed in tumor tissue, with significant prognostic value for tumor stage. The prognostic power of ANLN was demonstrated by qRT-PCR (n = 40) and immunohistochemistry (n = 209) on different cohort of patients in our institution. To further test the prognostic value of ANLN on an external cohort, we used public databases NCBI-GEO datasets (GSE31684, n = 93) and TCGA (n = 408). The expression level of ANLN mRNA was validated to be increased with tumor stage, and higher expression was indicative of poorer prognosis. To our knowledge, this is the first study to show a role of ANLN in BLCA. In fact, expression of ANLN has been found to be up-regulated in some other human tumors, with the exception of brain tumor^16^. Furthermore, Hall *et al*.^16^ found that ANLN expression showed a tumor progression-related pattern in breast, ovarian, kidney, colorectal, hepatic, lung, endometrial and pancreatic cancer. These observations were supported by several recent reports, which showed that elevated ANLN expression was associated with poorer overall survival of colorectal cancer^24^, breast cancer^25^, lung cancer^23^ and upper urinary tract urothelial carcinoma^26^. These observations indicated that ANLN expression was significant clinically relevant and might play an essential role in the pathogenesis of cancer. Above all, ANLN is a promising molecular biomarker for complementing clinicopathological classification for different risks of BLCA.

ANLN protein was first isolated from *Drosophila* embryo extracts by virtue of its binding affinity to F-actin^27^. As a key regulator of cytokinesis, it was not surprising that ANLN might play a critical role in carcinogenesis. However, the role and function of ANLN in BLCA remained unexplored. Through functional studies, we found that knockdown of ANLN could significantly inhibit the proliferation of BLCA both *in vitro* and *in vivo*. Furthermore, knockdown of ANLN strongly suppressed the migration and invasion ability of J82 and 5637 cells; GO analysis after ANLN knockdown also revealed that differentially expressed genes were mostly enriched in migration and locomotion in biological process. These results were consistent with clinical observation that ANLN expression was increased with higher stage of BLCA. These observations confirmed that ANLN played an important role in BLCA invasion and metastasis. Meanwhile, this finding was also in line with previous studies by Suzuki *et al*.^23^ and Zhou *et al*.^28^, who reported that ANLN participated in the proliferation and invasion of lung cancer and breast cancer, respectively. As we proved that ANLN-targeting siRNA was effective at inhibiting cancer cell growth and invasion, ANLN might become a candidate target for development of anticancer drugs. It was reported that carbon-ion beam irradiation effectively suppressed metastatic potential of lung cancer cells along with the reduced expression of ANLN, which indicated that expression level of ANLN could also be a potential maker for evaluation of cancer therapeutic efficacy^29, 30^. As patients with NMIBC have lifelong risk of recurrence and progression, ANLN could be a potential biomarker for stratifying the aggressiveness of BLCA.

Previous studies suggested that ANLN functions might be important in nuclear physiology, and dysregulation of ANLN might perturb the nuclei division in cancer^16^. In the present study, we could see significantly more multinucleate cells in morphologic observation after ANLN knockdown. Consistent with our observation, Engel *et al*.^31^ and Magnusson *et al*.^32^ reported that ANLN localization defect could induce formation of binucleate cells in cardiomyocyte cells and breast cancer cells, respectively. Moreover, we found cell cycle was arrested at G2/M phase in J82 and 5637 cells after ANLN knockdown, and less ANLN protein gathered around the nucleus to promote nucleus division in immunofluorescence analysis. Western-blot analysis suggested that the expression of cyclin B1 and D1 were decreased after ANLN knockdown, which could contribute to the G2/M cell cycle arrest. Meanwhile CCNB1, CENPF and BUB1B genes were revealed to be positively co-expressed with ANLN in bladder cancer from three different datasets. The cyclin B1, encoded by CCNB1, was an important cell cycle checkpoint protein, which involved in the regulation of G2/M transition^22^. Dai *et al*.^20^ investigated the function of CENPF in hepatocellular carcinoma cells, and their results suggested that CENPF knockdown resulted in the cell cycle arrest at G2/M checkpoint by down-regulating cell cycle proteins cyclin B1. The BUB1B, which encoded bub1b protein, was undetectable in G1 phase but its expression reached peaks in G2/M phase, and BUB1B was found to play distinct roles in the mitotic checkpoint^21, 33^. Therefore, ANLN may play a role in the regulation of cell cycle progression, and the mechanisms of interaction between ANLN and other cell cycle related genes warrant further investigation.

RhoA was reported to interact with ANLN through the conserved C-terminal domain of ANLN, and this was essential for the function and localization of ANLN in cells^19, 34, 35^. The function of ANLN was also suggested to be regulated by phosphorylation in a cell cycle phase dependent manner. Suzuki *et al*.^23^ indicated that ANLN might be phosphorylated by PI3K/Akt pathway or some other unidentified signaling pathway(s). To the present, however, it is still not known what kinase(s) is directly responsible for ANLN phosphorylation^14^. We performed microarray analysis to identify potential mechanisms responsible for ANLN function. In addition to PI3K/Akt pathway, other dysregulated pathways, e.g. Jak-STAT, Toll-like receptor and TNF signaling pathways were also likely to be correlated with ANLN function. However, elucidation of the precise mechanisms by which ANLN phosphorylation was directly regulated requires further studies.

In conclusion, we identified ANLN through RNA-seq as a frequently overexpressed gene in bladder cancer, and proved that ANLN could serve as a single and highly predictive prognostic biomarker in BLCA by using both internal and public cohort validation. In addition, we revealed that ANLN played a crucial role in the carcinogenesis of bladder cancer by participating in the regulation of proliferation, migration, invasion and cell cycle progression in BLCA cells.

## Materials and Methods

### Patients and tissue samples

From June 2013 to May 2014, ten pairs of BLCA samples and corresponding noncancerous epithelial tissues were obtained for RNA-seq from 10 newly diagnosed patients at different stages and grades from Changhai hospital. Detailed clinical information of these patients was summarized in Supplementary Table S1. Two other sample collections for validation were collected by radical cystectomy or transuretheral resection of bladder tumor from the same institution from July 2008 to December 2015; 40 for quantitative real time polymerase chain reaction (qRT-PCR) and 209 for immunohistochemistry (Supplementary Table S2); none of these patients received chemotherapy or radiotherapy previously. Informed consent was obtained from all patients and the experimental protocols of all experiments involving human and animals were approved by the ethical board of Changhai Hospital and performed in accordance to corresponding approved regulations or guidelines.

### Next-generation sequencing of transcriptome

RNA-Seq was performed by Beijing Genomics Institute (Shenzhen, China). All these samples were collected from radical cystectomy and examined by pathologists to ensure that tumor samples selected had tumor density >80% and the adjacent normal tissues were without tumor contamination. Experimental procedures of RNA-Seq were described previously^36^.

### RNA extraction and qRT-PCR

Total RNA was extracted using TRIzol reagent (Invitrogen) per the manufacturer’s instructions, and cDNA was synthesized with the 1st strand cDNA Synthesis Kit (PrimeScript™, Takara). qRT-PCR using 40 cycles of THUNDERBIRD® SYBR® qPCR Mix (TOYOBO) was applied to quantify ANLN mRNA concentrations, and GAPDH mRNA was used for normalizing the mRNA level. The primers for qRT-PCR are listed in Supplementary Table S3.

### Immunohistochemistry and immunofluorescence

Formalin-fixed paraffin-embedded BLCA samples were cut into 5-μm-thick sections. Antigen retrieval and immunostaining of sections were performed as described previously^37^. Primary antibody (1:100) anti-ANLN was purchased from Abcam (ab154337). Staining intensity was classified in a blinded fashion by pathologists. The immunohistochemical stain was scored on the percentage of positively tumor cell nucleus (negative, score 0; <1/3, score 1; 1/3–2/3, score 2; >2/3, score 3) and the color intensity of cytoplasm (negative, score 0; stramineous, score 1; buffy, score 2; dark brown, score 3). The two scores were combined, scores of 0–3 were defined as low expression, and 4–6 were defined as high expression. Immunofluorescence was performed using a standard protocol as described previously^23^. Primary antibodies were as follows: anti-ANLN (1:100), anti-F-actin (1:100, Abcam, ab130935). Images of immunofluorescence were taken by confocal microscope (Leica).

### Database mining

Two publicly available databases: gene expression omnibus (GEO) datasets (http://www.ncbi.nlm.nih.gov/gds/) and The Cancer Genome Atlas (TCGA, https://tcga-data.nci.nih.gov/tcga/) were used for data mining. Data were analyzed using cBioPortal (http://www.cbioportal.org/) or R project.

### RNA interference and lentivirus construction

The specific small interfering RNA (siRNA) against ANLN (si-ANLN, sense 5′-GCAAACAACUAGAAACCAATT-3′, antisense 5′-UUGGUUUCUAGUUGUUUGCTT-3′) and non-specific siRNA (sense 5′-UUCUCCGAACGUGUCACGUTT-3′, antisense 5′-ACGUGACACGUUCGGAGAATT-3′) were purchased from GenePharma, Shanghai, China. Cells were cultured to 70–80% confluence and transfected with siRNA using Lipofectamine RNAiMAX (Invitrogen) according to manufacturer’s instructions. Small hairpin RNA against ANLN (designed based on siRNA sequence and listed in Supplementary Table S3) and scrambled control sequence was inserted into pHBLV-U6-ZsGreen-Puro lentiviral vectors (Hanbio, Shanghai, China). The recombinant lentivirus was produced by co-transfection of 293 T cells with lentiviral vectors, plasmids pSPAX2 and pMD2G with LipoFiterTM (Hanbio, Shanghai, China). Lentivirus-containing supernatant were harvested 48 h after transfection and filtered through 0.22-μm cellulose acetate filters (Millipore). Recombinant lentiviruses were concentrated by ultracentrifugation (2 h at 50,000× g).

### Cell proliferation, invasion and migration assays

BLCA cell lines used in this study were all purchased from the American Type Culture Collection. Cells were cultured in Roswell Park Memorial Institute 1640 medium (Gibco®) supplemented with 10% fetal bovine serum and 100 μg/ml penicillin-streptomycin in a 37 °C incubator supplemented with 5% CO_2_. Cell proliferation rate post siRNA transfection was calculated using CCK-8 assay kit (Dojindo) per the manufacturer’s instructions. Five replicate wells were tested per assay. For scratch migration assay, cells were scratched using a standard 10 μl pipette tip 6 hours after transfection with siRNA against ANLN or control siRNA. Serial photographs were taken at different time points using an inverted phase contrast microscope (Olympus) and Image J (https://imagej.nih.gov/ij/) was used to calculate the scratch area. Invasion was measured using 24-well BioCoat™ Matrigel® Invasion Chamber (Corning®, #354480). Cells (3 × 10^4^) were seeded in the upper compartment in serum-free medium 24 hours after siRNA transfection, and the lower chamber was filled with medium containing 15% fetal bovine serum. Cells invading through the matrigel coated inserts were fixed with absolute alcohol and stained by crystal violet after 24 hours’, 72 hours’ incubation for J82 and 5637 cells, respectively. Cells were counted at least 10 randomly fields per insert at 400x magnification. Each experiment in J82 and 5637 cell lines was performed in triplicate.

### Tumor formation in nude mice

All animal experiments were undertaken with the approval of the Scientific Investigation Board of the Second Military Medical University. For preparation of the subcutaneous model, 100 μl (PBS: matrigel = 2:1) J82 cells infected with lenti-shANLN or lenti-vector viruses were injected subcutaneously into the lower flank of female nude mice at a concentration of 1 × 10^7^ cells/ml (each group with 6 mice). The technique to establish intravesical orthotopic BLCA model were described in the previous study (each group with 4 mice)^38^. Tumor size was measured weekly and tumor volume was calculated using the following formula: Volume = Length × width^2^ × 0.52(mm^3^).

### Flow cytometry analysis

Cells were collected for flow cytometry analysis 72 hours after si-ANLN or control siRNA transfection. Cell cycle and apoptosis analysis were performed using cell cycle kit and apoptosis kit (Lianke Biotech Co., Ltd., China) per manufacturer’s instructions. Each set was repeated at least three times.

### Microarray analysis

The total RNA was extracted after J82 cells were transfected with siRNA against ANLN or control siRNA for 24, 48 and 72 hours, respectively. The Whole Human Genome Oligo Microarray for the three pair samples (Agilent Technologies) was done by KangChen Bio-tech (Shanghai, China). The microarray data have been submitted to the Gene Expression Omnibus public database following the Minimum Information About a Microarray Experiment guidelines. The accession number are GPL13497 (platform) and GSE81629 (samples).

### Statistical analysis

The statistical tests were performed either by R project or GraphPad Prism 5 (GraphPad Software, Inc., La Jolla, CA). Pooled results were presented as mean ± SEM. Student *t* test, Chi-square test or Fisher’s exact test were used to compare continuous parametric data and categorical data between two groups, respectively. Cancer-specific survival (CSS), progression-free survival (PFS) and recurrence-free survival (RFS) were analyzed using Kaplan-Meier survival curve and log-rank test. Univariate and multivariate Cox regression analysis were used to calculate hazard ratio of risk factors. Two-sided p-value < 0.05 was considered statistically significant.

## Electronic supplementary material

Supplementary Figures

Supplementary Table S1-4

Supplementary Table S5

Supplementary Table S6
