# Supplementary material for: Transcriptome sequencing identifies ANLN as a promising prognostic biomarker in bladder urothelial carcinoma
Source: Sci Rep. 2017 Jun 9;7:3151. doi: 10.1038/s41598-017-02990-9 (PMC5466664; doi:10.1038/s41598-017-02990-9)
Supplement: Supplementary file 1 — Supplementary Figures [file 41598_2017_2990_MOESM1_ESM.pdf]

Transcriptome sequencing identifies ANLN as a promising prognostic biomarker in bladder urothelial carcinoma

Authors: Shuxiong Zeng[1*], Xiaowen Yu[2*], Chong Ma[1*], Ruixiang Song[1], Zhensheng Zhang[1], Xiaoyuan Zi[1], Xin Chen[1], Yang Wang[3], Yongwei Yu[3], Junjie Zhao[1], Rongchao Wei[1], Yinghao Sun[1#], Chuanliang Xu[1#]

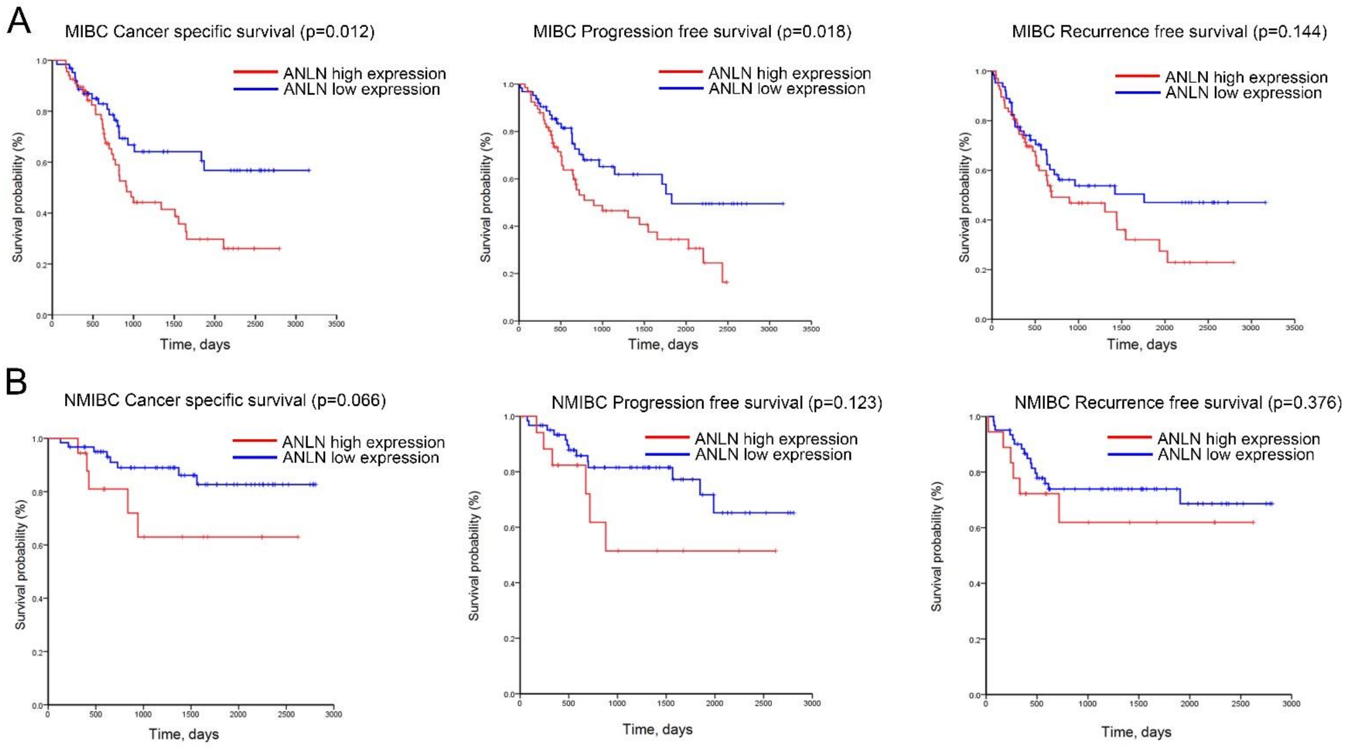

Figure S1. Kaplan–Meier survival curve comparing patients with different expression levels of ANLN. (A) Cancer specific survival, progression free survival and recurrence free survival curve between ANLN high and low expression muscle invasive bladder cancer (MIBC) patients. (B) Cancer specific survival, progression free survival and recurrence free survival curve between ANLN high and low expression non-muscle invasive bladder cancer (NMIBC) patients.

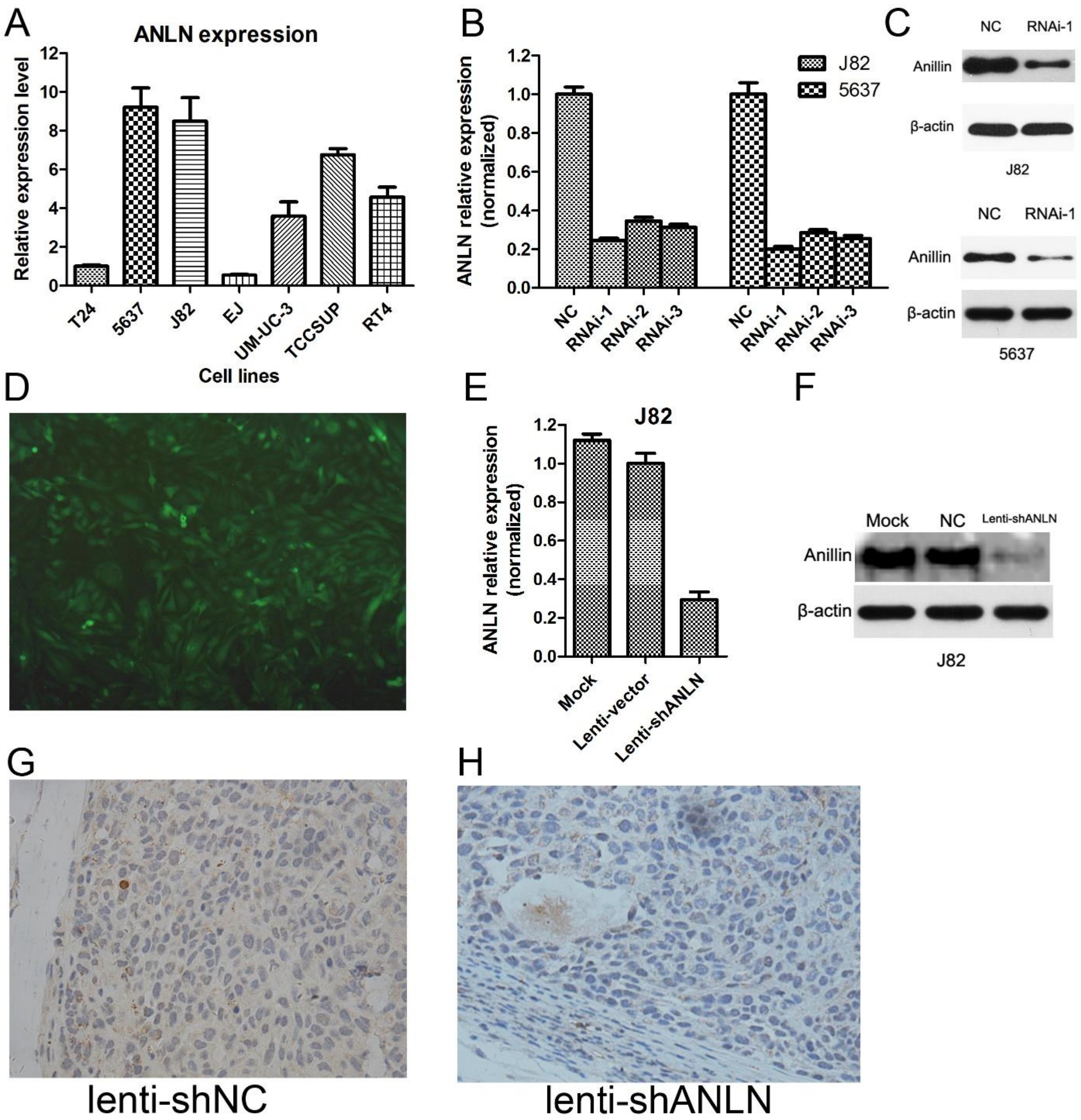

Figure S2. The efficiency of ANLN knockdown by si-ANLN and lenti-shANLN. (A) The expression level of ANLN mRNA in different bladder cancer cell lines. (B) Three different siRNA against ANLN were tested for knockdown efficiency, the most efficient one was used for further experiment. (C) Western-blot confirmed that ANLN expression could be significantly inhibited by si-ANLN. (D) Representative image of GFP fluorescence showed that J82 cells were successfully transfected with lenti-shANLN. (E-F) qRT-PCR and western-blot revealed ANLN expression was knockdown by lenti-shANLN. (G-H) Representative images of immunohistochemical staining of tumor tissues from xenograft mouse models showed that ANLN expression level was stably reduced by lenti-shANLN.

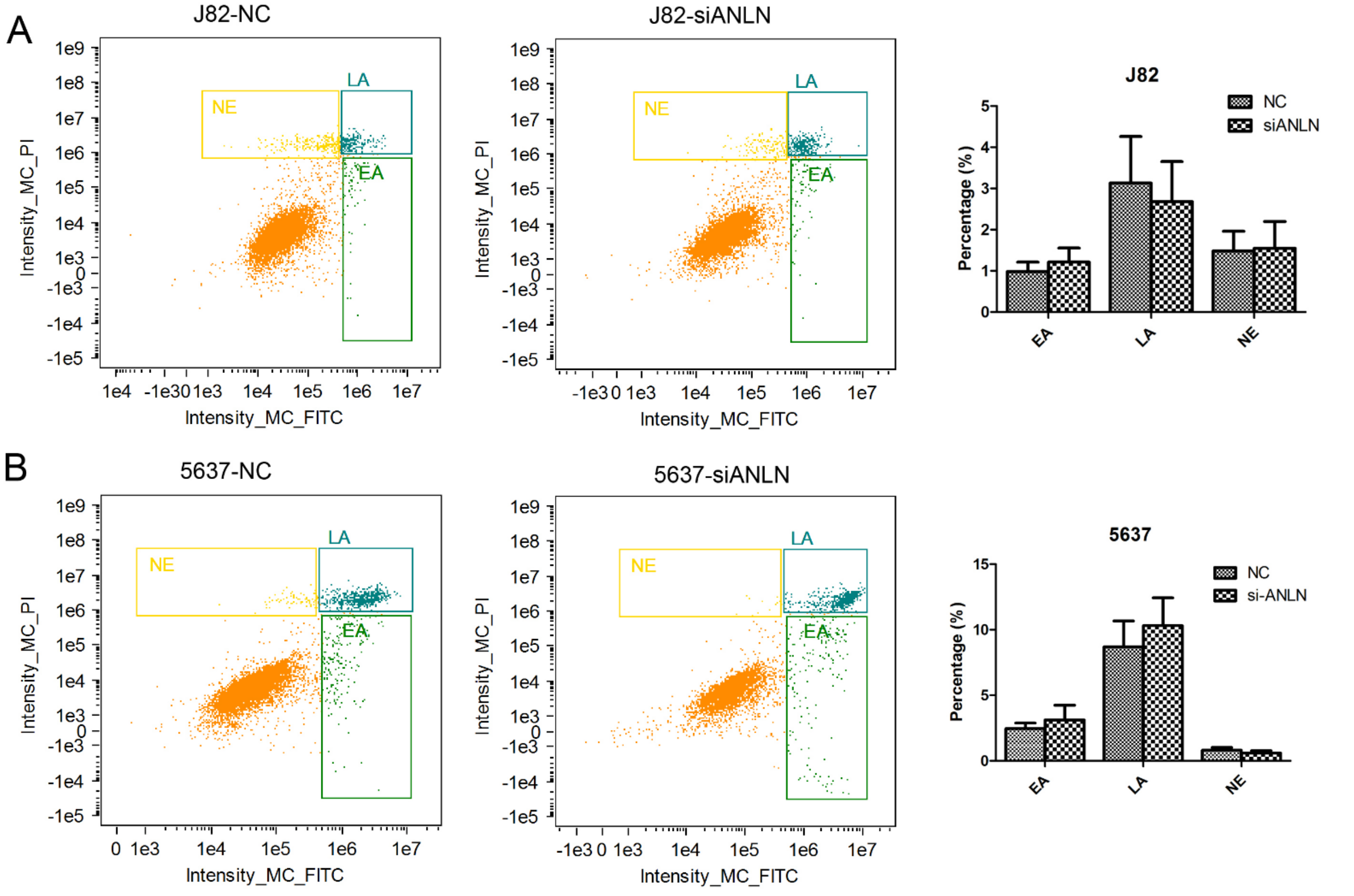

Figure S3. Apoptosis analysis using flow cytometry revealed no significant difference between ANLN knockdown group and control group. (A) Apoptosis analysis in J82 cells. (B) Apoptosis analysis in 5637 cells.

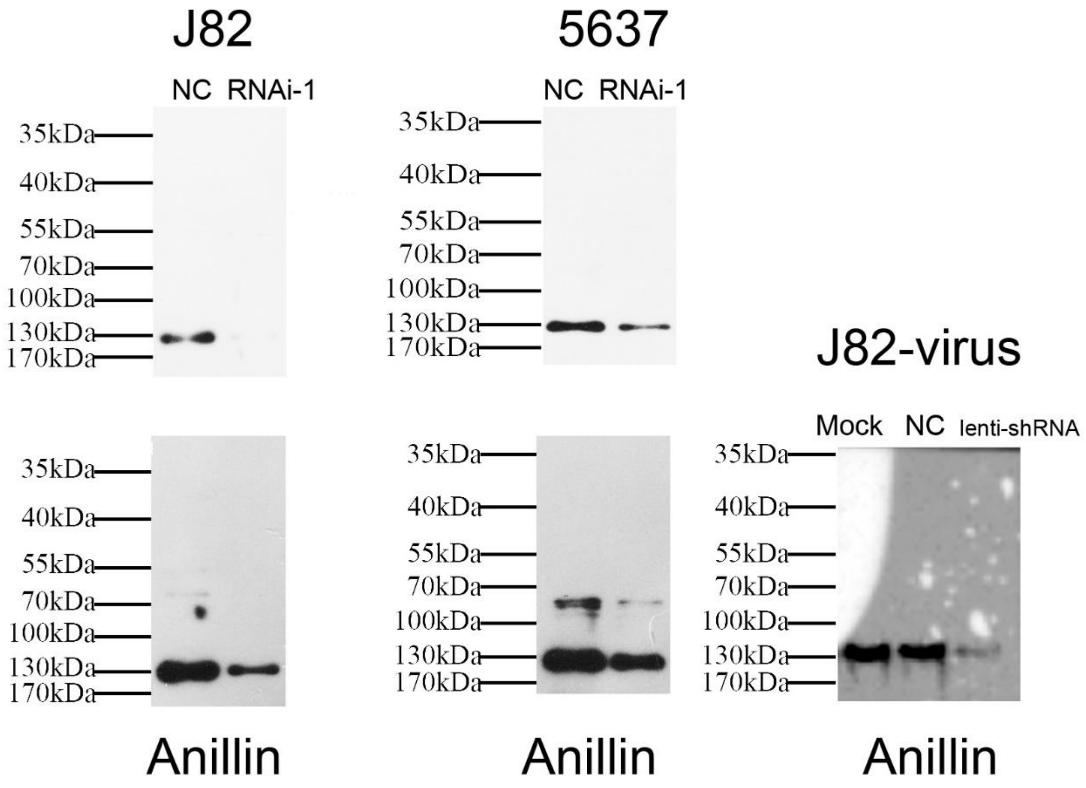

Figure S4. Raw data of western-blot analysis of Figure S2.

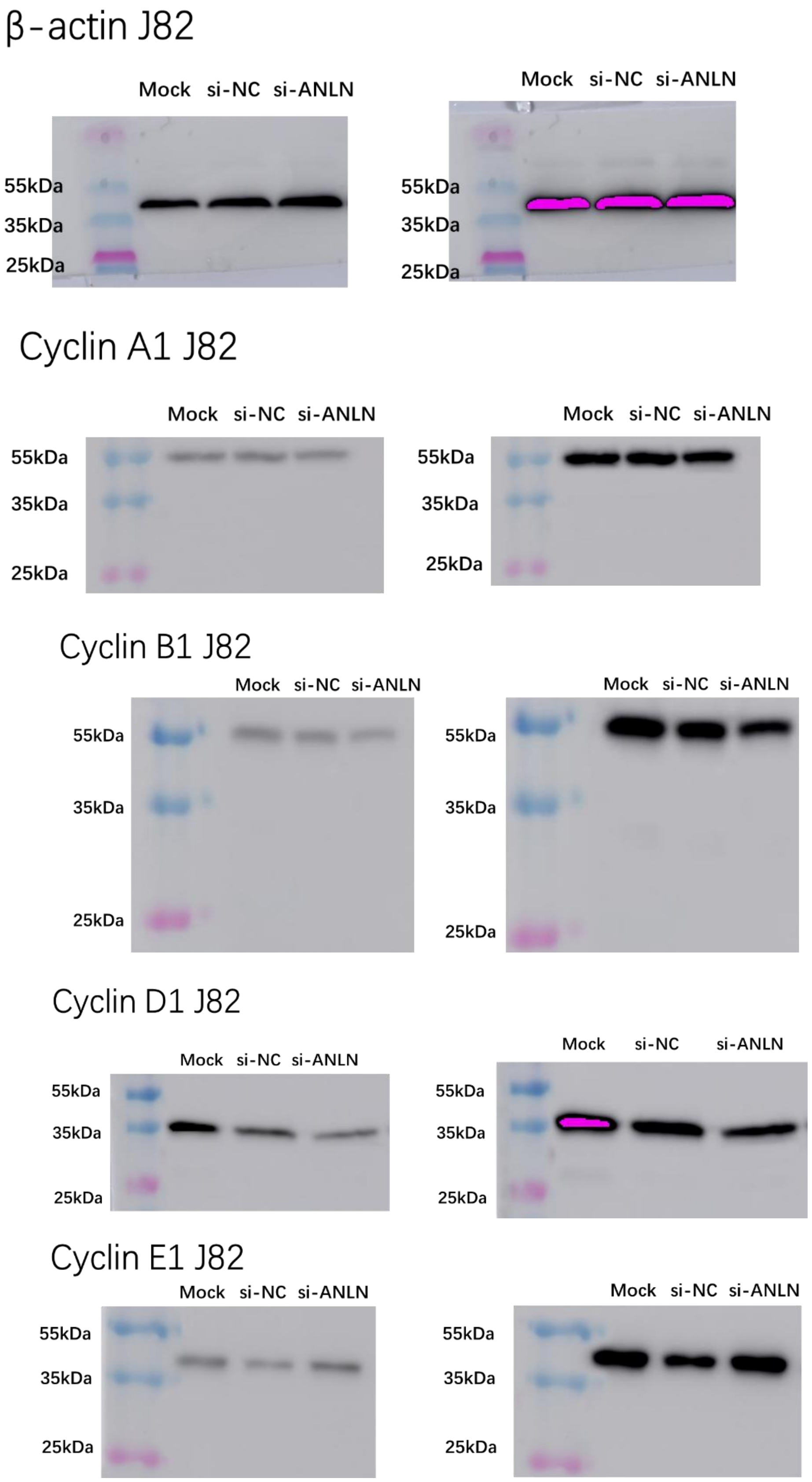

Figure S5. Raw data of western-blot analysis of cell cycle proteins expression in J82 cells with different treatments.

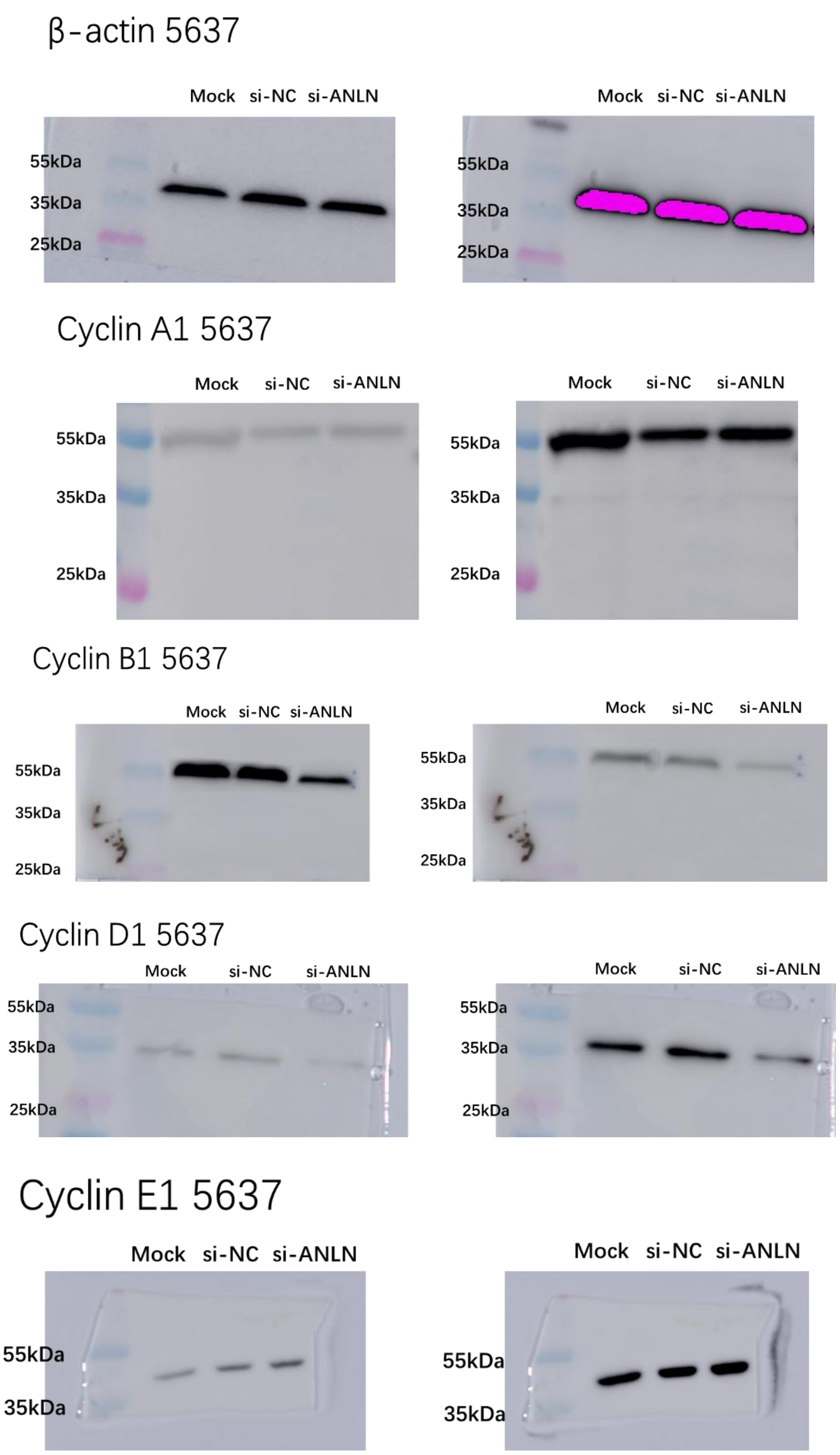

Figure S6. Raw data of western-blot analysis of cell cycle proteins expression in 5637 cells with different treatments.
