# Supplementary material for: Transcriptome sequencing identifies ANLN as a promising prognostic biomarker in bladder urothelial carcinoma
Source: Sci Rep. 2017 Jun 9;7:3151. doi: 10.1038/s41598-017-02990-9 (PMC5466664; doi:10.1038/s41598-017-02990-9)
Supplement: Supplementary file 2 — Supplementary Table S1-4 [file 41598_2017_2990_MOESM2_ESM.doc]

**Transcriptome sequencing identifies ANLN as a promising prognostic biomarker in bladder urothelial carcinoma**

**Authors:** Shuxiong Zeng^1*^, Xiaowen Yu^2*^, Chong Ma^1*^, Ruixiang Song^1^, Zhensheng Zhang^1^, Xiaoyuan Zi^1^, Xin Chen^1^, Yang Wang^3^, Yongwei Yu^3^, Junjie Zhao^1^, Rongchao Wei^1^, Yinghao Sun^1#^, Chuanliang Xu^1#^

**Table S1.** Clinical features of patients for transcriptome sequencing

| **Patient** | **Age (years)** | **Gender** | **Surgery type** | **Pathology** | **Grade** | **Chemotherapy history** |
| --- | --- | --- | --- | --- | --- | --- |
| 1 | 71 | Male | Cystectomy | T2N0M0 | High | No |
| 2 | 78 | Male | Cystectomy | T2N0M0 | High | No |
| 3 | 84 | Female | Cystectomy | T2N0M0 | High | No |
| 4 | 49 | Male | Cystectomy | T4N0M0 | High | No |
| 5 | 77 | Female | Cystectomy | T4N0M0 | High | No |
| 6 | 63 | Male | Cystectomy | T1N0M0 | Low | No |
| 7 | 69 | Female | Cystectomy | T1N0M0 | Low | No |
| 8 | 76 | Male | Cystectomy | TaN0M0 | High | No |
| 9 | 58 | Male | Cystectomy | TaN0M0 | High | No |
| 10 | 68 | Male | Cystectomy | TaN0M0 | High | No |

**Table S2** Baseline characteristics of patient for qRT-PCR and IHC validation

|  | **qRT-PCR cohort** | **IHC cohort** | **Tumor ANLN IHC level strong positive/(%)^*^** |
| --- | --- | --- | --- |
| **Age, median(IQR)** | 65 (56,70) | 69 (61, 75) | - |
| **Gender, men/women** | 31/9 | 144/65 | - |
| **Surgery type** |  |  | - |
| **TURBT** | 12 | 65 | - |
| **Cystectomy** | 28 | 144 | - |
| **Pathological stage** |  |  |  |
| **Ta, Cis, T1** | 19 | 79 | 18/79 (22.8) |
| **T2** | 13 | 70 | 29/70 (41.4) |
| **T3** | 6 | 54 | 33/54 (61.1) |
| **T4** | 2 | 6 | 5/6 (83.3) |
| **Grade** **(n%)** |  |  |  |
| **Low** | 14 | 51 | 8/51 (15.7) |
| **High** | 26 | 158 | 77/158 (48.7) |
| **Lymph node status** |  |  |  |
| **negative** | 38 | 198 | - |
| **positive** | 2 | 11 | - |

^*^ANLN expression was significantly higher in patients with muscle-invasive bladder cancer and high grade (p<0.001)

**qRT-PCR**=quantitative real time polymerase chain reaction; **IHC**=immunohistochemistry; **TURBT**=transurethral resection of bladder tumor; **IQR**=Interquartile range.

**Table S3**. Real time PCR primers and small hairpin RNA sequences

|  | **Forward 5’-3’** | **Reverse 5’-3’** |
| --- | --- | --- |
| **ANLN** | GCTGCGTAGCTTACAGACTTAGC | AAGGCGTTTAAAGGTGATAGGTG |
| **GADPH** | ACCACAGTCCATGCCATCAC | TCCACCACCCTGTTGCTGTA |
| **shRNA against ANLN** | GATCCGCAAACAACTAGAAACCAATTCAAGAGATTGGTTTCTAGTTGTTTGCTTTTTTC |  |
| **Non-silencing shRNA** | CTAGCCCGGCCAAGGAAGTGCAATTGCATACTCGAGTATGCAATTGCACTTCCTTGGTTTTTTGTTAAT |  |

**PCR**=polymerase chain reaction; **shRNA**= small hairpin RNA

| **Variables** | **Univariable**  **HR (95% CI)** | **P value** | **Multivariable**  **HR (95% CI)** | **P value** |
| --- | --- | --- | --- | --- |
| **Age (>65 vs.** ≦**65)** | 0.79 (0.48-1.29) | 0.342 | - |  |
| **Gender (male vs. female)** | 1.07 (0.63-1.79) | 0.810 | - |  |
| **Stage (<T2 vs. ≥T2)** | 3.35 (1.82-6.13) | <0.001 | 2.53 (1.33-4.8) | 0.005 |
| **Grade (high vs. low)** | 2.33 (1.19-4.56) | 0.014 | 1.21 (0.58-2.51) | 0.618 |
| **ANLN expression level (high vs. low)** | 2.75 (1.69-4.46) | <0.001 | 2.04 (1.21-3.43) | 0.007 |

**Table S4.** Univariate and multivariate cancer-specific survival Cox regression analysis

**HR**=hazard ratio; **CI**=confidence interval.
